# Transdiagnostic Clinical Staging for Childhood Mental Health: An Adjunctive Tool for Classifying Internalizing and Externalizing Syndromes that Emerge in Children Aged 5–11 Years

**DOI:** 10.1007/s10567-022-00399-z

**Published:** 2022-05-22

**Authors:** Vilas Sawrikar, Angus Macbeth, Karri Gillespie-Smith, Megan Brown, Andy Lopez-Williams, Kelsie Boulton, Adam Guestella, Ian Hickie

**Affiliations:** 1 Centre of Applied Developmental Psychology, University of Edinburgh, Edinburgh, UK; 2 ADHD & Autism Psychological Services and Advocacy, Utica, NY USA; 3 Brain and Mind Centre, University of Sydney, Sydney, Australia; 4 Present Address: Department of Clinical & Health Psychology, School of Health in Social Sciences, The University of Edinburgh, Medical School (Doorway 6), Room 1M.8, Teviot Place, Edinburgh, EH8 9AG UK

**Keywords:** Transdiagnostic mental health, Externalising problems, Internalising problems, Developmental psychopathology, Dimensional classification

## Abstract

Clinical staging is now recognized as a key tool for facilitating innovation in personalized and preventative mental health care. It places a strong emphasis on the salience of indicated prevention, early intervention, and secondary prevention of major mental disorders. By contrast to established models for major mood and psychotic syndromes that emerge after puberty, developments in clinical staging for childhood-onset disorders lags significantly behind. In this article, criteria for a transdiagnostic staging model for those internalizing and externalizing disorders that emerge in childhood is presented. This sits alongside three putative pathophysiological profiles (developmental, circadian, and anxious-arousal) that may underpin these common illness trajectories. Given available evidence, we argue that it is now timely to develop a transdiagnostic staging model for childhood-onset syndromes. It is further argued that a transdiagnostic staging model has the potential to capture more precisely the dimensional, fluctuating developmental patterns of illness progression of childhood psychopathology. Given potential improvements in modelling etiological processes, and delivering more personalized interventions, transdiagnostic clinical staging for childhood holds much promise for assisting to improve outcomes. We finish by presenting an agenda for research in developments of transdiagnostic clinical staging for childhood mental health.

Enhanced prevention, earlier intervention and delivery of more effective treatments for childhood-onset mental health problems represent an important agenda for tackling the “grand challenges” in mental health (Wakschlag et al., 2019). Mental disorders are among the leading causes of burden of disease among children globally and its relative contribution to disability is increasing compared to other conditions (Baranne & Falissard, 2018). High disease burden is further exacerbated by moderate rates of reliable improvement when children and families participate in evidence-based treatments in clinical settings (Ginsburg et al., 2014; Love et al., 2014; Nock, 2003). These challenges have called for investment in promoting prevention, early intervention and improving treatment effectiveness for childhood problems (Wakschlag et al., 2019). However, while remarkable progress has been made in promoting these clinical objectives, current approaches are poorly aligned to knowledge of individual developmental trajectories of mental health and psychosocial impairment in childhood (Colizzi et al., 2020). The Lancet Commission on sustainable development (Patel et al., 2018) identified clinical staging as a key mechanism for facilitating innovation in personalized and preventative care, and for promoting a lifespan approach, thus maximizing the benefits of early intervention.

Clinical staging was introduced by Fava and Kellner (1993) to align psychiatry with other areas of medicine. Clinical staging was proposed to enhance the utility of diagnosis by presenting a framework to improve the precision of treatment according to stage of illness while promoting early detection and secondary prevention in those presenting for care. Clinical staging has since been applied extensively to model the developmental course of several mental disorders in youth and adults (Cosci & Fava, 2013; McGorry & Hickie, 2019; Raouna et al., 2018). There are growing applications of clinical staging in personalizing care for these populations (Colizzi et al., 2020; Hickie et al., 2019a; Sawrikar et al., 2021; Shah, 2019). Interventions are stratified according to the likelihood of illness progression measured by clinical staging, indexing severity and chronicity, functional impairment, and neurobiological dysfunction (Hickie et al., 2019a). By contrast, research in applying clinical staging to childhood lags significantly behind (Sawrikar et al., 2021). Childhood is a period when mental health problems frequently emerge, and therefore opportunities for early intervention offer optimal propensity for adaptive change, alongside identifying early risk and enhancing environmental influences (Dadds & Frick, 2019).

In this article, we present a transdiagnostic staging model for childhood, alongside putative pathophysiological markers based on the tripartite model of common trajectories to mental illnesses in childhood (Hickie et al., 2019a; Scott et al., 2020). The article will first provide an overview of why a transdiagnostic staging model for childhood should be developed, reviewing transdiagnostic staging concepts and how they may apply to childhood psychopathology. Second, we present a model specifying staging criteria and pathophysiological mechanisms underlying trajectories toward internalizing and externalizing problems. Reasons for selecting internalizing and externalizing problems as exit outcomes of the staging model are discussed. Finally, we finish by proposing a research agenda to evaluating transdiagnostic clinical staging for childhood mental health.

## Current Relevance of a Transdiagnostic Staging Model for Childhood

Clinical staging in psychiatry emerged from criticisms of the deficits associated with current diagnostic systems for conducting research and developing effective treatments for common mental health problems (Hickie et al., 2013c; McGorry et al., 2006). Clinical diagnosis can be overly reductionistic, when based simply on the presence or absence of key symptoms. Typically, it does not capture complexities related to homotypic/heterotypic continuity and comorbidity (Shah, 2019). Moreover, the basis of diagnostic guidelines had been repeatedly questioned with criteria seemingly determined by committees largely relying on cross-sectional research drawn from adult cohorts (McGorry, 2019; McGorry et al., 2020). This is especially problematic when applied to younger age groups where early development of mental illness usually consists of non-specific symptoms that lack clear syndromal characteristics or boundaries (Hickie et al., 2019b). Patterns of illness progression among younger people are also noted to be probabilistic with not everyone progressing to full-threshold diagnosis. Diagnosis is therefore limited in its capacity to capture patterns of developmental psychopathology and personalized illness trajectories in younger people, with corresponding implications for lack of treatment specification (Linscott & Van Os, 2013; Shah et al., 2020).

Developments in clinical staging have occurred alongside other more dimensional approaches to enhanced classification. For instance, the Hierarchical Taxonomy of Psychopathology (HiTOP; Kotov et al., 2017) and Research Domain Criteria Initiative (RDoC; Insel et al., 2010) constitute two other emerging frameworks that utilize dimensional approaches. The HiTOP framework hierarchically arranges dimensions of mental health spectra (e.g., internalizing and externalizing) as high-order constructs, with mid-order syndromes/disorders and lower-order symptom components underneath, to capture covariation among symptoms within and across disorders (Conway et al., 2019). The RDoC framework proposed neurobiological substrates that may underpin the behavioral presentations of specific dimensions of brain function (Insel et al., 2010). Therefore, maladaptive functioning is conceptualized in relation to pathophysiology, with knowledge of dysfunction in neural circuits intended to inform targeted interventions.

**Fig. 1:**
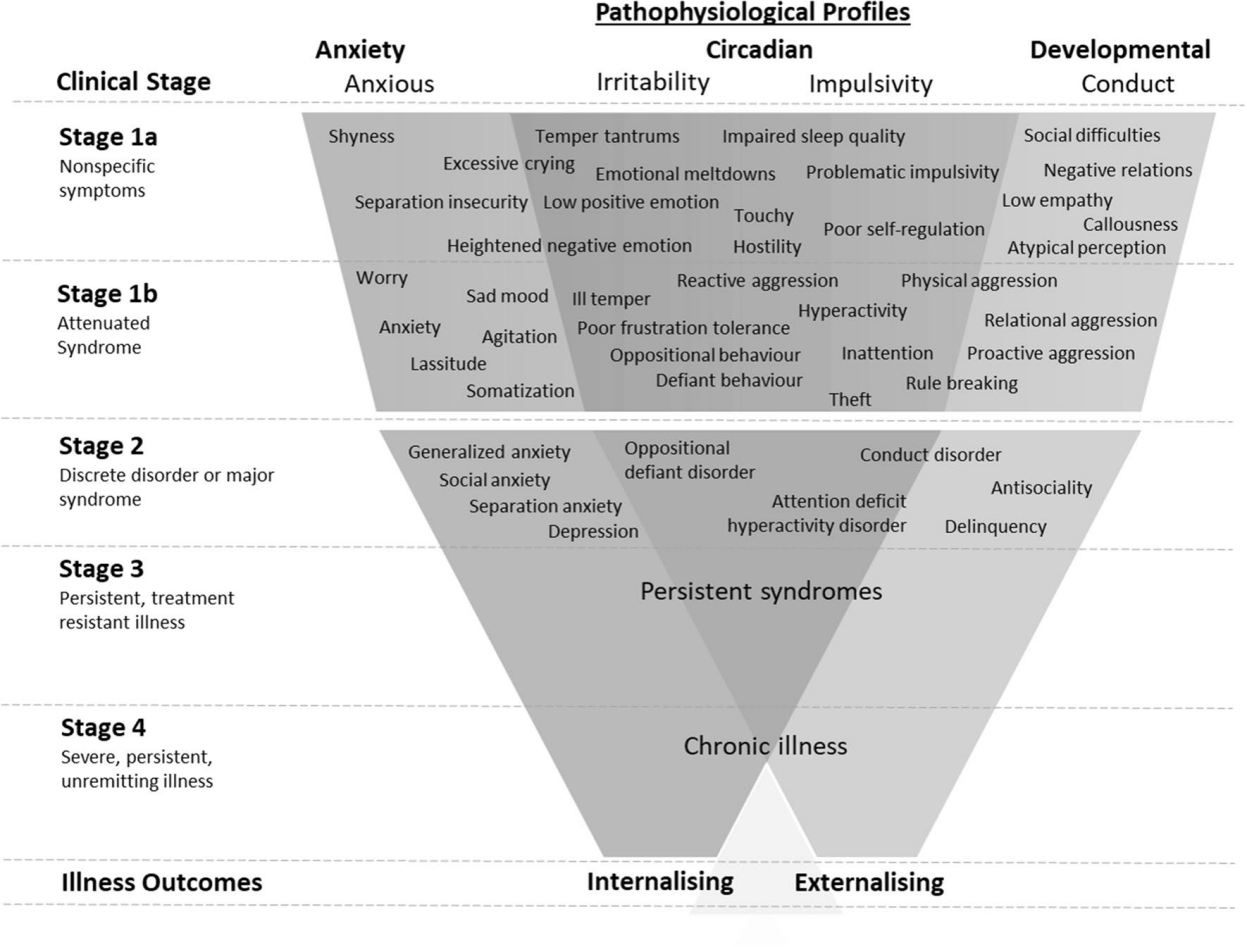
Transdiagnostic illness subtypes and pathophysiological profiles in trajectories to internalising and externalising syndromes in childhood. *Note* List of symptoms are exemplar only

By contrast, clinical staging proposed specific criteria for defining discrete stages of illness transition or progression (Fig. 1; see Cosci & Fava, 2013 for review). The early stages initially reflect milder and nonspecific clinical phenomena with lower risk of progression (Stage 1a) that then give way to attenuated syndromes that are more likely to precede transitions to full-threshold syndromes (Stage 1b). Subsequent stages capture progressed illness reflecting full-threshold syndromes, characterized by clinically significant severity and impairment (stage 2) and probable progression to recurrent, persistent, and treatment resistant forms of illness (stage 3 +). Transdiagnostic staging models apply a developmental science framework by integrating knowledge of developmentally vulnerable periods of life and mechanisms in risk for illness progression. For instance, clinical staging criteria within the tripartite youth model specify illness subtypes and pathophysiological mechanisms in psychosis, anxiety, and mood syndromes emerging in adolescence (12–25 years; Hickie et al., 2019a). The tripartite model describes the fluid course of unique and overlapping symptomatology that wax and wane in expression over time, allowing for a “pluripotent” approach to specifying illness subtypes and the identification of transdiagnostic and specific risk factors for major mental disorders (Carpenter et al., 2019; Hartmann et al., 2021). Evaluation of criteria have provided preliminary support for organizing biological, psychosocial, and neuroscientific research findings into a framework for treatment selection and outcome prediction (e.g., Hickie et al., 2019b; McGorry et al., 2014, 2018).

Applications of clinical staging in clinical care aim to personalize care for individual’s current and future needs (Shah et al., 2020). This is done through stratifying treatment according to clinical stage, illness subtype(s), and related pathophysiology (Rohleder et al., 2019; Salagre et al., 2018). For instance, stratifying treatment by clinical stage improves the logic and timing of interventions by facilitating treatment selection by staged risk/benefit criteria (Hartmann et al., 2021; Mei et al.,^2019^). Cross and colleagues showed that individuals in earlier stages show better response to simpler treatment regimens, which translates into less intensive, aggressive, and safer treatment approaches for those with milder presentations (Cross et al., 2016, 2017). By contrast, research shows that more intensive care is required for individuals in later clinical stages (Salagre et al., 2018). Higher intensity treatment entails integrated care approaches, alleviating current symptoms and risk, while targeting longer-term functional improvement to prevent further illness progression (Colizzi et al., 2020; Sawrikar et al., 2021). Stratification according to illness type and pathophysiology adds another layer of treatment personalization by aiding in identifying and targeting neurobiology underlying pathological processes. This has the potential to optimize treatment response by aligning treatment and preventive intervention according to individual disease processes accounting for comorbidity, severity, and functional impairment (Ramaswami et al., 2018).

Considering these developments, it is equally crucial to contemplate the importance of transdiagnostic staging criteria for conditions that have their onset in childhood, and particularly in the period from 5 to 11 years of age. Current research suggests that many childhood mental health problems, particularly those characterized as internalizing and externalizing disorders, are dimensionally distributed in the population, with risk states and subthreshold levels preceding full-threshold disorders (Flouri et al., 2019; Goodman et al., 1998; Norén Selinus et al., 2016; van Os, 2013). Childhood-onset problems are also one of the most reliable predictors of negative psychosocial outcomes in adulthood alluding to the propensity for progression in maladaptive functioning across the life course (e.g., Bevilacqua et al., 2018; Copeland et al., 2015). Indeed, half of all lifetime disorders have their onset during childhood, highlighting 5–11 years of age as a vulnerable period for the onset of lifetime mental health difficulties (Kessler et al., 2005). As the probability that childhood mental disorders will progress or persist over time are influenced by cumulative and interacting biological and social determinants of mental health, there are clear opportunities for early intervention and secondary prevention by targeting early risk and protective factors (Beauchaine, 2015; Gluckman et al., 2016).

There is also growing recognition of the importance of identifying transdiagnostic risk factors across the major disorders in childhood (Boulton et al., 2021; Dadds & Frick, 2019). Comorbidity is the rule rather than the exception, with approximately half of children with one diagnosable disorder also having a second disorder (Egger & Angold, 2006; Wichstrøm et al., 2012). This comorbidity increases with developmental delays, with most children showing multiple psychological disorders. There is also evidence of heterotypic continuity whereby childhood behavioral problems prospectively predict the onset of affective problems during transitions from childhood to early adolescence (Copeland et al., 2013). Consequently, evidence for comorbidity and heterotopy among children points to potentially shared causal processes in line with pluripotential conceptions of illness subtypes, further highlighting limitations in the utility of traditional diagnostic silos (Shah et al., 2020). Transdiagnostic models aim to account for comorbidity with particular attention to neural circuits that shape mental health outcomes (Barch, 2017; Beauchaine & McNulty, 2013; Dadds & Frick, 2019; Levy, 2010). For instance, neurodevelopmental models of child psychopathology emphasize partly overlapping reciprocal interactions between those neural subsystems that underlie transdiagnostic risk for psychopathology (Beauchaine & McNulty, 2013; Dadds & Frick, 2019; Levy, 2010). It is proposed that childhood-onset disorders are best understood in terms of underlying neuroprogression, with that dimension then predicting the future course of illness (Du Rietz et al., 2021; Shaw et al., 2010; Wakschlag et al., 2018).

A further rationale for a fully transdiagnostic staging model for childhood mental health is its clinical utility in enhancing early detection, targeted intervention, and secondary prevention. Identification of early signs of childhood mental illness may be difficult given as various behaviors may simply be assessed as age appropriate (e.g., tantrums or fear of the dark; Daniels et al., 2012; Langley et al., 2002). However, clinical staging distinguishes typical versus atypical development by specifying early risk markers and other features that are informed by severity, illness type, and functional impairment and disability that signal risk for progression prior to onset of diagnosable illness. For instance, atypical development may be identified even at stages 0 or 1a if pathophysiological signatures can be identified, in which individuals with indicators of biological risk may benefit from additional screening and monitoring leading to primary prevention prior to signs of syndromal development (Colizzi et al., 2020). For children presenting at stage 1b or higher, secondary prevention targeting functional impairment, pathophysiology and determinants of illness trajectory associated with illness type may be used to inform clinical care to reduce the risk of chronic and persistent illness (Rohleder et al., 2019). Staging and early intervention in childhood in turn optimizes the potential for recovery and arresting illness progression across the lifespan (Sawrikar et al., 2021).

Finally, recognizing transdiagnostic risk for mental illness among children with very early neurodevelopmental phenotypes (e.g., autism, pervasive developmental disorders) is crucial to determining treatment needs in childhood. Early neurodevelopmental syndromes are often lifelong, associated with atypical cognitive, social and emotional development from the first years of life (Boulton et al., 2021). Consistent with views of neurodiversity, however, early developmental divergence is not always representative of disordered behavior requiring intervention (Sonuga-Barke & Thapar, 2021). Instead, research emphasizes transdiagnostic developmental risk for affective, behavioral, and functional impairments in understanding mental health needs concomitant with neurodevelopmental conditions (Boulton et al., 2021). For instance, children with early neurodevelopmental conditions are three to six times more likely to have a comorbid diagnosis compared to their peers, with most common being internalizing and externalizing disorders (Einfeld et al., 2011; Salazar et al., 2015). Further, neurocognitive deficits manifest in a transdiagnostic manner and may represent a mechanism underlying functional impairment and disability in early neurodevelopmental syndromes (Kavanaugh et al., 2020; Klein et al., 2012).

Recently, clinical staging criteria for childhood affective syndromes were presented in applying clinical staging to stepped care approaches in mental health (“staged care”; Sawrikar et al., 2021). Whilst this model represents a step forward, the complete array of vulnerability was not considered, giving impetus for consideration of a transdiagnostic childhood staging model. For instance, reasons for referral to clinical services include disruptive behavior and neurodevelopmental disorders alongside affective syndromes (Olfson et al., 2014; Smith et al., 2018), all of which have peak onset in childhood (Kessler et al., 2005). Further, there are gender differences in the prevalence of affective and behavioral problems which are themselves correlated with neurobiological and socio-environmental factors. (Kramer et al., 2008). Importantly, the tripartite model recognizes emotional and behavioral difficulties as early risk markers for lifetime mental disorders. Therefore, clinical staging criteria without reference to affective and behavioral syndromes potentially obscures the breadth and overlap in developmental patterns for mental health problems in clinically referred children.

## Transdiagnostic Clinical Staging Model for Childhood

The transdiagnostic staging model for childhood mental health presented in this paper extend the criteria in Sawrikar et al. (2021) to include internalizing outcomes representing affective syndromes and externalizing outcomes representing behavioral syndromes. It is based on the tripartite model applied to youth mental health which has been extensively evaluated (Hickie et al., 2019b). The criteria are adapted for the management of internalizing and externalizing syndromes with considerations to clinical severity, functional impairment and disability, and chronicity. Notably, very early onset neurodevelopmental phenotypes or syndromes are recognized as major risk factors (Stage 0) for the onset of both internalizing and externalizing syndromes. Differences in disability and functional impairment are assessed within each stage as indicators of need for intervention and risk for chronic illness.

Reasons for selecting internalizing and externalizing syndromes as exit endpoints are first discussed. The current model follows the HiTOP dimensional framework that proposes the optimal meta-structure of psychopathology places disorders underneath internalizing and externalizing spectra allowing dimensions to covary within and across illness types (Conway et al., 2021). This meta-structure is naturally transdiagnostic and explicitly addresses comorbidity in classification (Krueger & Eaton, 2015). Importantly, these dimensions are appropriate for measuring emerging psychiatric symptoms in childhood with evidence that peak onset for these syndromes occur between 5 and 11 years of age (Kessler et al., 2005). Finally, the developmental course of these syndromes is consistent with probabilistic progression in clinical staging. Both conditions are characterized by early nonspecific symptoms that evolve to more clearly defined syndromes over time which have the propensity to persist or predict lifetime mental disorders (Copeland et al., 2009; Fanti & Henrich, 2010; Sterba et al., 2007). However, both conditions may also remit and/or be ameliorated by treatment (Comer et al., 2013; Compton et al., 2002).

Table 1 and Fig. 1 outlines the main features of the transdiagnostic model for staging internalizing and externalizing illness trajectories in childhood (5–11 years), from risk to end stage disease. The childhood staging model is underpinned by the assumption that the child’s risk for mental illness is influenced by biological and social determinants of mental health (Table 2; Sawrikar et al., 2021). These determinants are specified in Stage 0 (“at-risk: no current symptoms”) capturing known individual, family/caregiver, and social/environmental risk factors for the onset of internalizing and externalizing symptoms. However, Stage 0 risk factors can also operate concurrently increasing risk for transitions to higher stages (Iorfino et al., 2019; Shah, 2019). The model proposes that illness progression partly results from how environmental risk factors interact with biological vulnerabilities in perpetuating comorbidities and risk for chronic illness (Allott, 2019; Reiss, 2013). Thus, environmental risk factors represent potentially modifiable treatment targets to prevent stage transitions or illness progression and improve prognosis (e.g., parenting, family environment, daily functioning; Sawrikar et al., 2021).

**Table 1:** Transdiagnostic criteria for clinical staging of internalising and externalising syndromes emerging in childhood (5–11 years)

Clinical stage	Disability and functioning	Internalising and/or externalising symptoms
Stage 0: at-risk—no current symptoms	No impairments	No current symptoms
Stage 1a: nonspecific symptoms	Mild to moderate impact on social, educational, physical, and daily living	Mild to moderate severity without specific features indicative of more disabling syndromes
Stage 1b: attenuated syndrome	Moderate to severe impact on social, educational, physical, and daily living	Moderate severity with specific symptoms indicative of attenuated syndromes
Stage 2: discrete disorder or major syndrome	Severe and ongoing impact on social, educational, physical, and daily living	Meets criteria for internalising and/or externalising disorder
Stage 3: persistent, treatment resistant illness	Ongoing impact on social, educational, physical, and daily living lasting at least 2 years or over a 12-month period after entry into psychological, pharmacological, or multidisciplinary intervention	Symptoms lasting at least 2 years, with ≤ 3 months of remission *or* no improvement at 12 months after entry into psychological, pharmacological, or multidisciplinary intervention
Stage 4: severe, persistent, unremitting illness	Evidence of marked deterioration in social, educational, physical, and daily living due to persistence illness	Chronic symptoms lasting at least 5 years *or* no improvement after 2 years after entry into psychological, pharmacological, or multidisciplinary intervention

Refer to Table 2 for Stage 0 risk factors

**Table 2:** Psychosocial and biological risk factors in Stage 0 for childhood internalising and externalising syndromes

Type of risk	Risk factor
Individual	Early onset neurodevelopmental phenotype or syndrome; Perinatal injury; Prenatal conditions; Temperamental risk factors; Atypical social and cognitive profiles; Language difficulties; Motor skills delay; Social or learning difficulties at school transition; Poorer physical health; Child abuse or neglect
Family/caregiver	Emotional distress (e.g., depression/ anxiety) of primary caregiver; Caregiver instability/ unstable family environment; Parental conflict or relationship dissatisfaction; Style of parenting (e.g. inconsistent or harsh discipline); Loss of a parent or other grief/ illness in the family or close social network; Family history of mental ill health; Unemployment of parent who is primary earner; Material conditions (access to resources, food/nutrition, water, sanitation, housing, employment)
Other social and environmental	Financial hardship; Disadvantaged neighbourhood Community based participation; Violence/ crime; Access to and quality of local services

Stage 1 represents the earliest presentations of illness, split into Stage 1a—nonspecific symptoms and Stage 1b—attenuated syndromes (Cosci & Fava, 2013). Stage 1a captures nonspecific symptoms representing mild clinical symptoms, as well as mild to moderate impairment in functional domains. Stage 1a criteria emphasizes behavioral and emotional traits associated with internalizing and externalizing phenotypes, distinguishing risk from normal variation (Kotov et al., 2017). Stage 1b captures moderately severe internalizing and externalizing but ‘attenuated’ syndromes with moderate to severe impairment in functional domains (McGorry et al., 2018). This is identified by specific symptoms of internalizing (e.g., anxiety, sadness, and somatization) and/or externalizing syndromes (e.g., oppositionality, defiance, hyperactivity), with functional impairments in one or more environments (e.g., home, school, and cocurricular) reflecting attenuated syndromes with higher risk for stage progression.

A critical cut off or stepwise transition in the model exists at the point when the child meets criteria for Stage 2 and higher. Stage 2 represents a more discrete transition into signs of full-threshold syndromes with concomitant greater likelihood of persistent or further illness progression (Carpenter et al., 2019). Stage 2 criteria emphasize higher clinical severity and ongoing and major impairments in functional domains occurring in multiple environments that warrant intensive and longer-term care (Hermens et al., 2013; Tickell et al., 2019). In Stages 3 and higher, criteria emphasize treatment resistance, longer periods of illness, and persistent functional impairment in addition to Stage 2 criteria. As with other illness progression models, it is important to note that as distinct from clinical ‘state’ (where remission and recovery are possible at any stage), individuals are not classified as returning to prior clinical stages after receiving effective treatment (Hickie et al., 2013b).

## Proposed Pathophysiological Mechanisms

The aim is to develop a staging framework that links emerging clinical phenotypes to neurophysiological mechanisms, both transdiagnostically and those that may be unique to internalizing and externalizing problems. The goal is to promote treatments that may target relevant neurophysiological substrates (Insel et al., 2010; McGorry et al., 2006). Our model proposes describing individual illness trajectories by putative illness subtype(s) based on assessments of clinical phenotype and related pathophysiology, and that these subtypes represent the common profiles of internalizing/externalizing trajectories. The proposed illness subtypes for childhood build on the tripartite model presented for early onset mental disorders (5–25 years), that detail three common profiles: anxious-arousal, circadian dysregulation, and developmental (Fig. 1; Hickie et al., 2013c; Scott et al., 2020). Recognizing emerging and dynamic clinical phenotypes that link to these profiles is aimed at furthering investigation of those developing neural systems that underpin the internalizing and externalizing syndromes that are common in childhood. The illness profiles focus on those neural circuits involved in emotion functioning and social learning (e.g., Activation, Integration, Discrimination, Response and Reward (AIDRR) circuits, DeMayo et al., 2019) proposed to explain variations in internalizing and externalizing etiology and comorbidity (Drabick et al., 2010; Tucker et al., 2015). Deficits in systems modulating these circuits are also emphasized adding to the proposed neurophysiological basis of illness profiles (Beauchaine, 2015).

The first illness subtype (‘anxious—internalizing’) follows the anxious phenotype and hyperarousal pathophysiology in illness trajectories to affective syndromes. For our model, this relates to fear-related and distress-related internalizing problems in childhood (Achenbach & Rescorla, 2001). The anxious phenotype captures hyperarousal processes of heightened sensitivity (i.e., stress-reactivity) to fear and threat (Hickie et al., 2013a, 2013b, 2013c). Neural substrates relate to altered functionality in the septohippocampal region, which include neural fear and emotion circuitry (e.g., amygdala activity) and stress-related responses, as well as deficient prefrontal inhibition of amygdala activity involving ventrolateral, ventromedial, and anterior cingulate subdivisions within the prefrontal region (e.g., Beauchaine & Zisner, 2017; Gold et al., 2016; Kujawa et al., 2016; Monk et al., 2008; Tang et al., 2019).

The second (‘irritability—internalizing/externalizing’) and third (‘impulsivity—externalizing’)’ illness subtypes that are presented are aligned, in part, to the circadian (24-h sleep–wake, activity and feeding rhythms) profile. The irritability subtype is based on evidence that trait irritability is a transdiagnostic risk factor for externalizing problems, depression/anhedonia, and mood lability in bipolar depression (Leibenluft & Stoddard, 2013; Zisner & Beauchaine, 2016). The impulsivity subtype is based on evidence that trait impulsivity confers liability for hyperactive–impulsivity, a vulnerability for all types of externalizing problems (Beauchaine & Zisner, 2017). These subtypes emphasize interactions between early difficult temperaments, 24-h sleep–wake and activity disruptions, mood lability, and affective and behavioral dysregulation, in determining transdiagnostic illness trajectories (Heiler et al., 2011). More specifically, the circadian system is proposed to have a role in the pathophysiology of internalizing-externalizing syndromes, with symptoms (i.e., mood dysregulation, impulsivity-hyperactivity) either representing the cause or consequence of sleep disturbances (Bijlenga et al., 2019; Carpenter et al., 2021). Neural circuits connecting the central circadian network, midbrain, and prefrontal substructures are hypothesized to underly the circadian profile. Namely, ineffective modulation of deficient dopaminergic substructures from subdivisions of the orbitofrontal and dorsolateral prefrontal cortex, alongside other circuits that link the suprachiasmatic nucleus, hypothalamic nuclei and pineal gland, manifest deficits in self-regulation characteristic of the circadian profile (Carpenter et al., 2021).

Cases of emerging externalizing symptoms in context of low empathy, and related deficits in social learning and higher order functioning are allocated to a fourth developmental subtype (Carpenter et al., 2019). This subtype relates to the conduct phenotype in the tripartite model (Miranda et al., 2017; Scott et al., 2020), referring to impairments of social cognition in unique trajectories to antisocial behavour among children with early signs of autistic and callous-unemotional traits (Blair, 2013; Georgiou et al., 2019; Pasalich et al., 2014). Empathy deficits are proposed to impair instrumental learning and moral development needed for learning prosocial behavior (Baumeister & Lobbestael, 2011; Frick et al., 2014). Attention is given to differences in theory of mind and emotion recognition, attention, and responsiveness, with a focus on cortical regions in the temporal lobe associated with the social perception, as well as fronto-limbic pathways mediating motivational and emotional responses to socially salient stimuli (Dadds & Frick, 2019; DeMayo et al., 2019).

It is important to note that the putative illness subtypes do not represent mutually exclusive pathophysiological pathways. For instance, children are expected to demonstrate non-specific symptoms that may cut across illness types in earlier clinical stages (van Os, 2013). At later stages, the child is expected to show clearer phenotypic expression, functional impairment, and more specific neurobiological correlates (Carpenter et al., 2019). Further, children’s illness type may relate to multiple atypical neural circuits. For instance, the presence of trait anxiety may attenuate the severity of impulsive behavior in provocative situations as children are likely to take pause before reacting (Beauchaine et al., 2017). Conversely, children with trait impulsivity could experience worse outcomes if a low anxiety temperament (e.g., callous-unemotional traits) is evident as these children show diminished avoidance of aversive cues (e.g., Anderson & Kiehl, 2014; Blair, 2010; Viding et al., 2012). Finally, children may shift between pathways over time showing heterotypic comorbidity, whereby children who may initially align to one phenotype (e.g., impulsivity) in early clinical stages progress to symptoms overlapping components from another phenotype (e.g., anxiety) in later stages (Hartmann et al., 2021). These aspects of homotypic, heterotypic, and comorbid trajectories warrant empirical attention for childhood conditions.

## Research Agenda for Staging Childhood Mental Health Problems

**Fig. 2:**
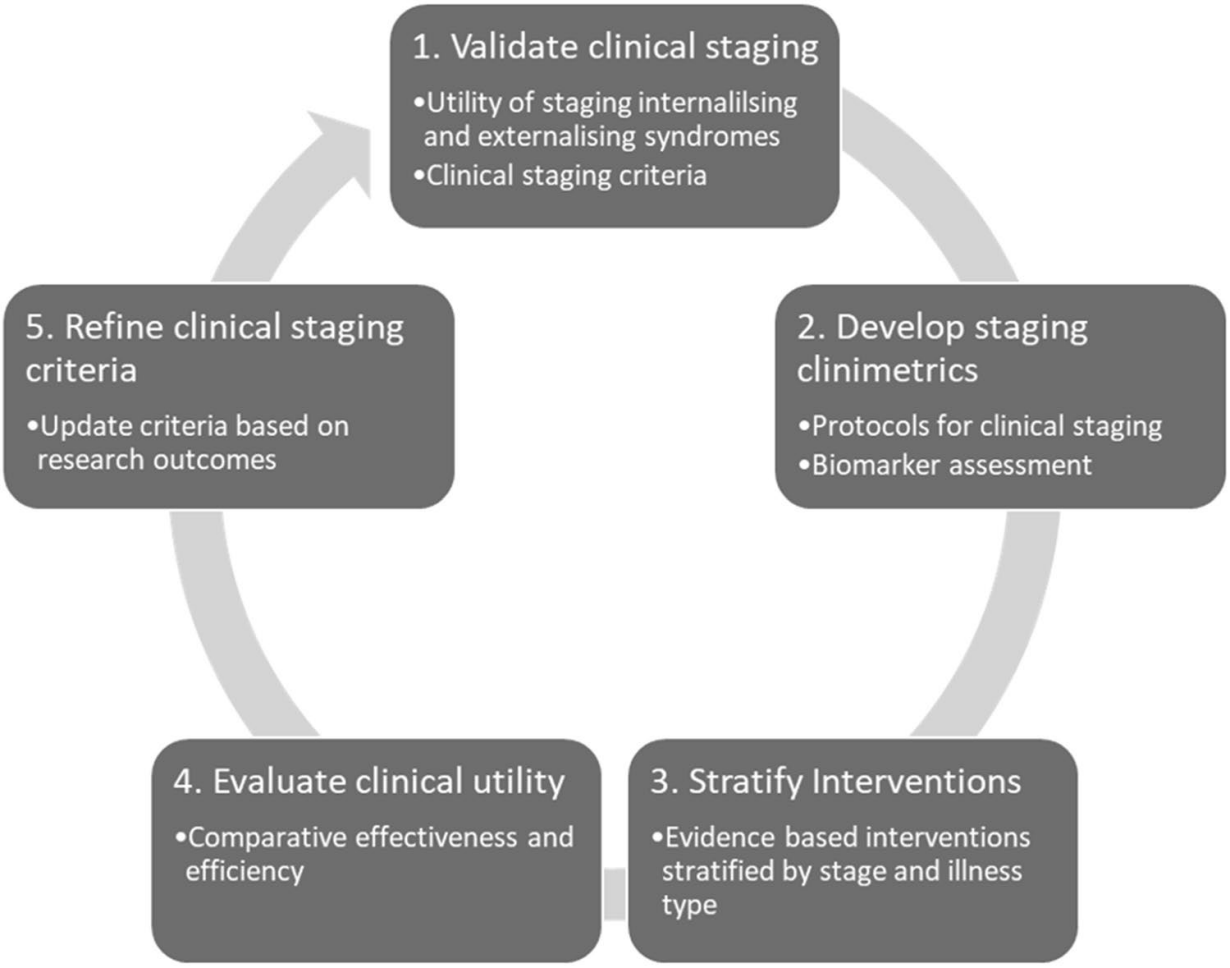
Research agenda to progress transdiagnostic clinical staging for internalizing and externalizing syndromes that emerge in children aged 5–11 years

The transdiagnostic staging criteria for childhood presented in this article represent a preliminary model. It adapts criteria established for youth to childhood building upon previously published criteria (Sawrikar et al., 2021). The criteria are tentative and should be continuously redeveloped in response to research findings. Our proposed research agenda, summarized in Fig. 2, is designed to highlight key areas of development.

### Validate Staging Criteria

There is need to validate proposed staging criteria for childhood, via testing the reliability of clinical stage assessment, monitoring longitudinal course, and evaluating its predictive utility for treatment selection. Specific areas for future research include examining associations between clinical stage, individual characteristics, and subtypes of illness trajectories, as well as the role of clinical stage in influencing treatment processes and outcomes. Based on previous research, we hypothesize that clinical stage criteria would show high internal consistency and validity supported by evidence that clinical stages are associated with graded clinical severity, distress, functional impairments, and neuropsychological profiles (Cross et al., 2016; Iorfino et al., 2019; Scott et al., 2013). Likewise, previous studies have examined the validity of illness subtypes by asking which biopsychosocial characteristics are strongly associated with each subtype, and which combination of characteristics distinguish illness subtypes (Hickie et al., 2013a, 2013b, 2013c). These should be examined in relation to clinical staging of various internalizing and externalizing syndromes.

### Develop Clinimetrics Protocols for Assessment

Assessment protocols for clinical staging children will need to be established as required information is not included in existing diagnostic approaches (i.e., clinimetrics; Fava et al., 2018). Comprehensive assessment of patterns of symptoms, severity of illness, comorbid conditions, timing of onset, rate of illness progression, and responses to previous treatments, are all required to demarcate children into clinical stages (Fava et al., 2011). Thus, research should seek to determine which measures of symptoms, functionality, and mental health are optimal for staging childhood internalizing and externalizing syndromes. Emerging protocols that focus on domains of childhood mental health, functioning and quality of life, caregiver mental health, cognitive functioning, and family background may have significant utility in harmonizing protocols for clinical staging (Boulton et al., 2021). These protocols emphasize multidimensional assessments using measures that have good psychometric properties, normative data, and sensitivity to detect change from clinical interventions.

### Identify Biomarkers of Illness Type and Clinical Stage

Characterization of pathophysiological mechanisms will benefit from additional detection of biomarkers that correlate with clinical stages. Defined biomarkers (i.e., measurable biological characteristics) may then augment clinical staging and enhance assessment of types and extent of disease progression in internalizing, externalizing, and comorbid trajectories (McGorry et al., 2014). While the identification of specific biomarkers in mental health is still highly problematic, (Peterson, 2020), various frameworks propose domains of neuroinflammation, oxidative stress, lipid metabolism, neuroanatomical structure, hypothalamic–pituitary–adrenal axis, and chronobiology as key areas (e.g., McGorry et al., 2014). Profiling based on such domains of biomarker measurement help define each child in a way that promotes more personalized intervention (Salagre et al., 2018).

### Define Staged Care Treatments for Childhood

Significant gaps exist in research to guide decisions of stage appropriate treatments. The best empirically supported treatments (ESTs) for childhood mental health problems are tied to single clinical diagnosis, while treatments for children in earlier clinical stages and/or presenting with nonspecific symptoms are less established (Shah et al., 2020). Therefore, there is an urgent need for the development of treatment approaches that align with contemporary thinking on the developmental etiology underlying illness progression, as proposed by the clinical staging framework.

Following our model, we recommended building an evidence base of interventions stratified by clinical stage and illness subtype, and taking into account individual clinical and biological characteristics underlying disease progression (Manchia et al., 2020). Treatments could target-specific biobehavioural features of temperamental vulnerability or adapting already existing ESTs to address neurocognitive deficits/excesses (Dadds & Frick, 2019; McClowry et al., 2008). Given that neuroadaptive sensitivity to environmental influence is high during childhood (Colizzi et al., 2020), we advocate for increased investment into psychosocial interventions that address the interplay between neurodevelopmental vulnerabilities and adverse environments, to ensure the safest but effective front-line treatment options are available for children. Family based treatments, for instance, represents a well-established EST for child and family maladjustment known to have long-term benefits for child cognitive and emotional development (Beauchaine et al., 2005; Kaminski & Claussen, 2017; Webster-Stratton et al., 2011). Further, family based treatments may be adapted to target unique neurodevelopmental profiles of children classified by trait-based clinical phenotypes (e.g., CU traits; Kimonis et al., 2019). Where front-line treatment options are ineffective, clinical staging would pave the way for sequential treatment schedules with increasing intensity in response to treatment non-response.

### Evaluate the Clinical Utility of Staged Care

Staged care needs to be formally evaluated for its comparative effectiveness compared to treatment as usual. At the individual level, attention should be given to evaluating whether stratifying treatment selection based on stage and illness type results in better clinical outcomes compared to those based on a conventional diagnosis. Outcomes should include assessment of changes in symptoms and distress, as well as the ability of stage-based interventions to prevent illness progression to advanced stages (McGorry et al., 2006). At population level, evaluation entails examining whether stage care in childhood has the potential to improve accessibility/ equity, acceptability/ satisfaction, efficiency/ expenditure/ cost, effectiveness/ outcomes, appropriateness and care continuity/ coordination, while ultimately reducing the occurrence of mental health difficulties within childhood and over the life course (Sawrikar et al., 2021).

### Global Sustainable Development in Using Clinical Staging

The final suggestion circles back to the initial premise of this article: sustainable development in mental health (Patel et al., 2018). Substantial attention has been given to reforming mental health care to include prevention alongside intervention to reduce the global burden of disease associated with mental illness (Institute of Medicine 2006). In line with this, clinical staging has the potential to guide population health-oriented systems of care providing promotive, preventive, and curative services, as well as ensuring adequate provision for rehabilitation and long-term care (Sawrikar et al., 2021). However, current research into applications of clinical staging is generally limited in scope, i.e., investigating the validity and utility of staging in youth or adult mental health, in clinical care settings, in well developed countries. Global sustainable development in using clinical staging requires aligning it with a convergence model of mental health, recognising evidence from research examining developmental, biological, and social determinants of mental health (Patel et al., 2018). For instance, an ongoing need exists for integrating global perspectives in achieving sustainable development goals. Social environmental factors may have a substantial role in determining prognosis in child mental health in low-middle income countries through differences in gene-by-environment interactions and epigenetic mechanisms or impacts on treatment (Compton & Shim, 2015; World Health Organization, 2017). Such considerations to social determinants of mental health are overlooked in clinical staging so far (Shah et al., 2020). We suggest that integrating social and biological factors in risk stratification as presented in Tables 1 and 2 could profoundly help in optimizing clinical staging in childhood mental health.

Further consideration of how clinical staging may operate across the life course warrants specific attention. Life course approaches to mental disorders is one of the main principles of global sustainability in mental health by emphasising the interplay of social and biological risk factors during key developmental stages over the life span (World Health Organization, 2014). To that end, there is need to clarify how childhood and youth mental health staging models might interact in order to progress a life course approach. For instance, Shah et al. (2020) ask whether childhood-onset disorders should be treated as a separate track of conditions, whether they represent risk states for youth-onset conditions, or both. We propose that ‘both’ is most appropriate to understanding the interface between childhood-onset and youth-onset mental health syndromes. By presenting criteria for childhood mental health, we posit childhood-onset conditions should be treated in their own right, and then assessed at any life course stage, to ensure individuals access the right level of care (Sawrikar et al., 2021). However, we agree that childhood-onset conditions that cause liability for new youth-onset mental disorders should also be recognized as risk states in the youth staging model, thus providing comprehensive case identification. We propose that decisions to stage clinical features as either a childhood-onset or new youth-onset condition relies on the assessment of continuity of illness subtype(s) which represent the common profiles of mental disorders emerging in individuals aged 5–25 years (i.e., anxious-arousal, circadian dysregulation, developmental). Our proposal is speculative at this stage as the methods for determining continuity (clinimetrics) require further explication.

## Conclusion

Childhood is a developmentally sensitive period where vulnerability for the onset of lifetime mental health difficulties is significant (Kessler et al., 2005). Conversely, opportunities for early intervention and secondary prevention are optimal during childhood (Colizzi et al., 2020). Developments in clinical staging can potentially improve classification and identification of treatment need, treatment selection, and further embed prevention in healthcare, while optimizing early intervention outcomes (Hickie et al., 2013b; McGorry et al., 2006).

Given potential improvements in modelling etiological processes, transdiagnostic clinical staging for childhood holds much promise in optimizing the effectiveness of treatment and prevention for children. Staging risk and prodromal features of childhood psychopathology in earlier clinical stages helps to identify those children that require earliest provision treatment to prevent the onset of clinical impairment (Colizzi et al., 2020). Secondary prevention becomes important for children at later stages presenting for care (Cross & Hickie, 2017; Hickie et al., 2019b). In improving clinical effectiveness, clinical staging that considers illness type and related pathophysiology could pave the way for developing targeted interventions alleviating underlying illness drivers (Rohleder et al., 2019). Based on these considerations, clinical care in mental health would benefit from researchers evaluating the utility of staging mental health and developing staged care treatments for childhood. The research agenda presented in this article will hopefully promote a new phase of development toward personalizing care for children based on clinical staging.

## Data Availability

No data were generated or analysed in the preparation of this article.
